# Pembrolizumab-Associated Adrenal Insufficiency Presenting as Adrenal Crisis: A Case Report

**DOI:** 10.7759/cureus.118142

**Published:** 2026-10-18

**Authors:** Laura Samet, Zainab Ali, Habib Haider

**Affiliations:** 1 Department of Emergency Medicine, University Hospitals of North Midlands NHS Trust, Stoke-on-Trent, GBR; 2 Department of Acute Medicine, University Hospitals of North Midlands NHS Trust, Stoke-on-Trent, GBR

**Keywords:** adrenal crisis, adrenal insufficiency (ai), immune checkpoint inhibitor (ici), immune-related adverse event (irae), immunotherapy toxicity, non-small cell lung carcinoma (nsclc), pembrolizumab immunotherapy

## Abstract

Immunotherapy regimens containing the programmed cell death protein 1 (PD-1) inhibitor pembrolizumab are widely utilised in the management of non-small cell lung cancer (NSCLC). However, the administration of pembrolizumab is associated with unique immune-related adverse events. Adrenal insufficiency is a rare, potentially life-threatening endocrinopathy that can manifest even after the cessation of planned treatment. A male in his 50s with metastatic pulmonary adenocarcinoma developed an acute adrenal crisis 12 days after completing a 24-month course of pembrolizumab immunotherapy. Routine outpatient blood tests first identified critical hypocortisolaemia (morning serum cortisol <50 nmol/L), prompting the patient to attend hospital. The patient subsequently developed severe hypotension (58/45 mmHg) and reported malaise, anorexia, and vomiting after oral intake. Septic shock was considered but felt to be less likely given the absence of infective symptoms. The patient was successfully resuscitated in the emergency department with administration of fluids and high-dose intravenous hydrocortisone. Following clinical stabilisation, under the supervision of the endocrinology team, he was transitioned to a long-term oral maintenance regimen consisting of hydrocortisone and fludrocortisone. This case illustrates that pembrolizumab-associated adrenal insufficiency can present as an abrupt, life-threatening crisis in the immediate post-treatment phase. Frontline clinicians must remain vigilant for late-onset endocrinopathies, regardless of the treatment completion date. Proactive patient education and long-term biochemical surveillance are imperative to ensuring early clinical intervention and avoiding catastrophic management delays and poor clinical outcomes.

## Introduction

Annually, approximately 50,200 new cases of lung cancer are diagnosed in the United Kingdom, making it the third most common cancer nationally [1]. Approximately 84% of all lung cancers are non-small cell lung cancer (NSCLC) [2], which carries an average five-year survival rate of 18.1% in the United Kingdom [1]. Pembrolizumab is an immune checkpoint inhibitor that has improved outcomes for patients with advanced malignancies, including NSCLC. It acts selectively upon programmed cell death protein 1 (PD-1) to restore anti-tumour T-cell activity. However, this non-specific immune activation is associated with a spectrum of immune-mediated adverse side effects, including pruritus, colitis and various endocrinopathies [3]. Although adrenal insufficiency remains rare, an emerging number of cases emphasise the potentially life-threatening nature of adrenal insufficiency secondary to pembrolizumab treatment. We present one such case in which a patient with NSCLC was identified as having adrenal insufficiency two weeks after completing a 24-month course of pembrolizumab therapy through timely clinical recognition in the acute emergency setting.

This article was previously presented as a meeting abstract and an oral presentation at the 2026 Association of Pakistani Physicians of Northern Europe (APPNE) CME Health Seminar on 10 October 2026.

## Case presentation

A man in his 50s with no prior comorbidities initially presented with a new-onset seizure, leading to the discovery of an intracranial mass. Subsequent staging investigations identified the primary pathology as metastatic pulmonary adenocarcinoma. He underwent induction therapy with four cycles of pembrolizumab, pemetrexed and carboplatin. This was followed by maintenance therapy with pembrolizumab and pemetrexed. From cycle 23 onwards, pemetrexed was discontinued due to tolerability issues, and pembrolizumab monotherapy was maintained until planned treatment cessation at cycle 35, completing a two-year course (Table 1). Twelve days after completing his final cycle, he was contacted because routine outpatient blood tests had shown critical hypocortisolaemia. He was initially advised to attend the oncology emergency assessment unit; however, due to acute bed constraints, he was redirected to the Emergency Department.

**Table 1:** Clinical timeline and treatment schedule. An in-depth breakdown of the patient's 35-cycle chemo-immunotherapy schedule and the timeline of the subsequent acute presentation. BP, blood pressure; ED, emergency department; IV, intravenous; NaCl, sodium chloride

Timeframe/Phase	Therapy regimen/clinical status	Key parameters and clinical notes
Induction phase (Cycles 1-4)	Pembrolizumab + Pemetrexed + Carboplatin	Administered every three weeks. Tolerated with expected treatment profile.
Early maintenance (Cycles 5-22)	Pembrolizumab + Pemetrexed	Dual-therapy maintenance regimen following induction.
Late maintenance (Cycles 23-35)	Pembrolizumab monotherapy	Pemetrexed stopped due to tolerability issues. Planned treatment cessation at cycle 35.
Day 11 post-treatment	Outpatient blood tests	Routine blood tests revealed critical hypocortisolaemia. Morning serum cortisol <50 nmol/L (reference range: 170-540 nmol/L).
Day 12 post-treatment (ED presentation)	Emergency department assessment	Patient reported a five-day history of malaise, anorexia and vomiting. Severe hypotension on examination (BP 58/45 mmHg). Remained alert and oriented. Passive leg raise improved BP to 81/50 mmHg. Septic shock excluded.
Day 12 post-treatment (management)	Emergency resuscitation and treatment	Administered 100 mg IV hydrocortisone stat and two 1-litre fluid boluses (0.9% NaCl). BP stabilised at 94/62 mmHg, with a rapid return of appetite.
Inpatient monitoring (Days 12-13)	Acute medical unit admission	IV hydrocortisone 100 mg every 6 hours was started. After a 24-hour period of stability, the patient was transferred to oral hydrocortisone (10 mg in the morning, 5 mg at noon and 5 mg in the afternoon) and fludrocortisone 100 mcg daily.

Upon assessment in the emergency department, the severe hypocortisolaemia was found to be acutely symptomatic. His blood tests from the previous day at 09:49 am (11 days post-treatment cessation) showed a morning serum cortisol level of <50 nmol/L (normal reference range: 170-540 nmol/L). The patient had not received exogenous steroids in the 18 months before the critical hypocortisolaemia reading. The patient reported a 5-day history of malaise, anorexia and persistent vomiting with oral intake. Initial observations showed significant hypotension with a blood pressure (BP) of 58/45 mmHg. Despite this critically low BP, he remained fully alert and oriented. He was immediately moved to a monitored space, where a passive leg raise manoeuvre marginally improved his BP to 81/50 mmHg. A septic pathology was considered; however, given the lack of pyrexia or focal infective symptoms, it was felt to be less likely. The clinical presentation was determined to be indicative of an adrenal crisis likely secondary to immunotherapy. The emergency team administered a stat dose of 100 mg intravenous hydrocortisone alongside aggressive fluid resuscitation, and the oncology team was urgently consulted.

Following discussion with the oncology team, the patient was deemed too haemodynamically unstable for transfer to the oncology ward and was subsequently admitted to the higher-monitoring unit under the acute medical team. Following the administration of two 1-litre fluid boluses of 0.9% sodium chloride and the initial corticosteroid dose, his BP stabilised at 94/62 mmHg. At this time, the patient reported a notable symptomatic improvement and a return of appetite. He was formally admitted under the acute medical team, with endocrinology team input for further diagnostic and therapeutic guidance. He was initially placed on a maintenance regimen of intravenous hydrocortisone 100 mg every 6 hours.

The endocrinology team agreed that the presentation was in keeping with acute adrenal insufficiency likely secondary to pembrolizumab immunotherapy. They advised a minimum 24-hour course of intravenous corticosteroid therapy before transitioning to an oral maintenance regimen. The maintenance regimen included hydrocortisone (10 mg in the morning, 5 mg at noon and 5 mg in the afternoon), alongside fludrocortisone 100 mcg once daily in the morning, as per the endocrinology team's advice. The patient responded well to treatment, with rapid stabilisation of vital signs and a swift return of appetite within 24 hours.

A comprehensive pituitary-adrenal hormone panel was requested, including adrenocorticotropic hormone (ACTH), luteinising hormone (LH), follicle-stimulating hormone (FSH), thyroid-stimulating hormone (TSH) and free thyroxine (fT4). Unfortunately, the serum ACTH sample was rejected by the laboratory due to a lack of a designated sample and could not be analysed. The patient’s renal function had deteriorated slightly from baseline; his baseline creatinine was 130-140 µmol/L and baseline eGFR was approximately 50 mL/min/1.73 m². The available results are detailed in Table 2.

**Table 2:** Summary of laboratory results. Glucose, lactate, bicarbonate and osmolality are from the initial venous blood gas. All other blood results are from formal laboratory tests. TSH, thyroid-stimulating hormone; fT4, free thyroxine; FSH, follicle-stimulating hormone; IGF-1, insulin-like growth factor 1; LH, luteinising hormone; ACTH, adrenocorticotropic hormone; eGFR, estimated glomerular filtration rate

Test	Result	Reference range	Status
Cortisol	<50 nmol/L	170-540 nmol/L	Low
TSH	2.03 mIU/L	0.38-5.33 mIU/L	Normal
fT4	14.8 pmol/L	11.5-22.7 pmol/L	Normal
Prolactin	121 mIU/L	45-375 mIU/L	Normal
FSH	15.9 IU/L	1.4-18.1 IU/L	Normal
LH	23 IU/L	1.5-9.3 IU/L	Elevated
IGF-1	5 nmol/L	6-27 nmol/L	Low
ACTH	Unavailable	-	Not tested due to lack of dedicated sample
Sodium	135 mmol/L	133-146 mmol/L	Normal
Potassium	4.1 mmol/L	3.5-5.3 mmol/L	Normal
Urea	8.6 mmol/L	2.5-7.8 mmol/L	Elevated
Creatinine	160 µmol/L	59-104 µmol/L	Elevated
eGFR	42 mL/min/1.7 m^2^	-	Low
Glucose	6.8 mmol/L	3.1-7.0 mmol/L	Normal
Lactate	1.4 mmol/L	0.6-1.4 mmol/L	Normal
Bicarbonate	23.8 mmol/L	22-26 mmol/L	Normal
Osmolality	278.4 mOsm/kg	275-295 mOsm/kg	Normal
Haemoglobin	128 g/L	130-180 g/L	Low
White cell count	6.44 × 10^9/L	4.0-11.0 × 10^9/L	Normal
Neutrophil count	1.73 × 10^9/L	2.0-7.5 × 10^9/L	Low
Eosinophil count	0.38 × 10^9/L	0.04-0.40 × 10^9/L	Normal
Platelet count	185 × 10^9/L	150-450 × 10^9/L	Normal

Given the available test results, the patient was treated under the working diagnosis of isolated adrenal insufficiency leading to adrenal crisis, likely secondary to pembrolizumab immunotherapy. The ability to differentiate primary adrenal insufficiency (e.g. adrenalitis) from secondary adrenal insufficiency (e.g. hypophysitis or isolated ACTH deficiency) was compromised by the lack of an ACTH result. Further tests, such as testosterone, renin, aldosterone and adrenal autoantibodies, would also have been beneficial and improved diagnostic clarity. Unfortunately, the patient was discharged before a repeat ACTH test could be attempted. A CT of the thorax, abdomen and pelvis was performed during the inpatient stay, which showed stable right upper lobe opacity and no new suspicious abdominal or pelvic abnormalities. The endocrinology team recommended continuing long-term hydrocortisone and fludrocortisone therapy and arranged outpatient clinic follow-up to monitor clinical recovery and biochemical response.

## Discussion

Pembrolizumab is a monoclonal antibody immunotherapy that inhibits PD-1 on T cells [4]. Under normal physiological conditions, PD-1 and its ligands, PD-L1 and PD-L2, inhibit signalling pathways to balance T-cell activation. This balance is fundamental to allow the host immune system to clear malignant cells without causing immune-mediated collateral tissue damage to self-tissues [5]. Cancer cells can exploit this pathway to suppress local immunity by expressing PD-L1 and PD-L2, which bind to PD-1 receptors on cytotoxic T cells, triggering T-cell deactivation and allowing the tumour to evade immune surveillance [5]. Pembrolizumab binding to the PD-1 receptors on T cells blocks this inhibitory interaction, preventing T-cell deactivation and restoring host immune clearance capabilities [4].

Clinical trials have shown that pembrolizumab offers superior overall survival outcomes when compared with chemotherapy (30 months vs. 14.2 months) while reducing grade 3-5 treatment-related adverse events (31.2% vs. 53.3%) [6]. Because pembrolizumab functions by blocking the PD-1/PD-L1 axis, therapeutic efficacy positively correlates with biomarker expression; patients with a tumour proportion score of 50% or higher for membranous PD-L1 display high clinical response rates [7]. Consequently, pembrolizumab has been licensed for use across multiple malignancies, including colorectal, oesophageal, renal, head and neck cancers and, notably, metastatic NSCLC [8].

Despite its clear therapeutic benefits, clinicians must remain aware of potential treatment complications. Common side effects of immunotherapy include pruritus, anorexia, fatigue, thyroiditis and hepatic toxicity [3,7]. Less frequent but severe immune-related adverse events (irAEs) include hypophysitis, adrenalitis, type 1 diabetes mellitus and pneumonitis [3]. The most prevalent endocrinopathies associated with anti-PD-1 monotherapy are primary hypothyroidism and hyperthyroidism, at approximately 5.9% and 1%-4.7% of patients, respectively [9]. Isolated PD-1 blockade is traditionally associated with a very low rate (<1%) of hypophysitis and adrenal insufficiency (1%). This is notable when compared with anti-CTLA-4 immunotherapies like ipilimumab, which exhibit substantially higher rates of hypophysitis, ranging from 0.4% to 17% [9]. A literature review estimated the combined rate of hypophysitis and adrenal dysfunction from anti-PD-1 monotherapy at just 1% to 2% [10]. However, the incidence of severe irAEs increases most notably when combination strategies are used; combining anti-CTLA-4 and anti-PD-1 agents results in a 13.9% incidence of hypothyroidism and an 8% incidence of hypophysitis [9].

Although immunotherapy-related endocrinopathies remain rare, they frequently present with vague symptoms that can mimic advanced malignancy or systemic infection. A major clinical challenge is the broad timeframe in which these presentations occur, both during active cycles and long after treatment cessation. Presentations of adrenal insufficiency due to adrenalitis or isolated ACTH deficiency have been reported as early as six weeks into treatment [11] and as late as eight months following treatment cessation [12-13]. Our patient presented uniquely in the immediate post-treatment window, developing an adrenal crisis less than two weeks after completing his planned 24-month regimen.

The clinical spectrum of adrenal insufficiency ranges from mild, asymptomatic biochemical deviations to life-threatening haemodynamic collapse. While universally accepted diagnostic criteria for emergency settings are limited, it is primarily a clinical diagnosis guided by symptom recognition. Typically, patients present with absolute hypotension (systolic BP <100 mmHg) or relative hypotension (>20 mmHg drop from baseline parameters), showing rapid resolution of symptoms upon administration of glucocorticoid therapy [14]. Concomitant symptoms frequently include fatigue, nausea, anorexia, abdominal pain, vomiting and acute electrolyte disturbances. Unmanaged presentations can quickly progress to refractory shock, metabolic encephalopathy, coma and death [15]. Approximately 5% to 10% of patients with established adrenal insufficiency will experience an acute adrenal crisis, carrying a mortality rate of 0.5 per 100 patient-years [14,16].

Differentiating between primary adrenal insufficiency (such as adrenalitis) and secondary adrenal insufficiency (such as hypophysitis or isolated ACTH deficiency) is clinically challenging in the acute phase. Differentiation requires structured hormone evaluation, including serum ACTH, LH, FSH, cortisol, prolactin, growth hormone and thyroid function tests. The physiological hypothalamic-pituitary-adrenal (HPA) axis begins with the hypothalamic secretion of corticotropin-releasing hormone (CRH), which stimulates the anterior pituitary to release ACTH. ACTH subsequently prompts the adrenal cortex to synthesise and release cortisol. Rising systemic cortisol levels provide a negative feedback suppression loop on both the hypothalamus and anterior pituitary to suppress further release of CRH and ACTH [17].

When adrenal insufficiency is suspected, a comprehensive anterior pituitary panel should ideally be taken before the administration of exogenous corticosteroids; however, life-saving management must never be delayed whilst awaiting blood collection. Biochemically, primary adrenal insufficiency is characterised by elevated serum ACTH due to loss of negative feedback, whereas secondary adrenal insufficiency demonstrates inappropriately low or suppressed serum ACTH levels; both will present with low serum cortisol levels [15]. If the wider anterior pituitary hormones remain intact while ACTH is low, a diagnosis of isolated ACTH deficiency is supported. Hypophysitis can cause isolated ACTH deficiency, partial hypopituitarism or pan-hypopituitarism, with ACTH and thyroid-stimulating hormone deficiencies representing the most common central losses [9]. Unfortunately, immunotherapy-related endocrinopathies often lead to permanent endocrine deficiencies requiring lifelong treatment [18].

Crucially, the initial emergency management of an adrenal crisis does not require confirmation of the underlying ACTH aetiology. Prompt administration of parenteral corticosteroids is the definitive, life-saving intervention, typically initiated with an intravenous loading dose of 100 mg hydrocortisone. This loading dose is followed by a maintenance dose of 200 mg of hydrocortisone over 24 hours, administered either as an infusion or equally divided into six-hourly intravenous administrations [19]. Patients require a period of clinical stabilisation before being transitioned to an oral maintenance regimen, a process that should be coordinated with local endocrinology teams to establish safe long-term monitoring and outpatient follow-up.

## Conclusions

Pembrolizumab and related immune checkpoint inhibitors represent a life-changing development in oncological care, significantly improving patient survival outcomes. However, these therapies carry a risk of irAEs ranging from mild toxicities to life-threatening emergencies. An awareness of these potential complications, particularly potentially life-threatening endocrinopathies such as adrenal crisis, is critical for frontline clinicians working within acute and emergency settings. Patients undergoing or completing immunotherapy need detailed education regarding side effects and warning signs to enable prompt escalation to emergency services. Crucially, assessing clinicians should not dismiss the possibility of immunotherapy-induced adverse events based solely on the treatment completion date, as irAEs have been shown to occur both during and months after treatment completion. Close monitoring of the HPA and thyroid axes should persist well into the post-treatment surveillance phase to ensure early detection and treatment of dangerous endocrinopathies. Given the highly variable clinical presentation and temporal onset of these toxicities, further research is appropriate to define standardised, safe follow-up protocols for this growing patient population.
